# Network-based prediction of polygenic disease genes involved in cell motility

**DOI:** 10.1186/s12859-019-2834-1

**Published:** 2019-06-20

**Authors:** Miriam Bern, Alexander King, Derek A. Applewhite, Anna Ritz

**Affiliations:** Biology Department, Reed College, Portland, OR USA

**Keywords:** Semi-supervised learning, Functional interaction network, Schizophrenia, Autism, Cell motility

## Abstract

**Background:**

Schizophrenia and autism are examples of polygenic diseases caused by a multitude of genetic variants, many of which are still poorly understood. Recently, both diseases have been associated with disrupted neuron motility and migration patterns, suggesting that aberrant cell motility is a phenotype for these neurological diseases.

**Results:**

We formulate the Polygenic Disease Phenotype Problem which seeks to identify candidate disease genes that may be associated with a phenotype such as cell motility. We present a machine learning approach to solve this problem for schizophrenia and autism genes within a brain-specific functional interaction network. Our method outperforms peer semi-supervised learning approaches, achieving better cross-validation accuracy across different sets of gold-standard positives. We identify top candidates for both schizophrenia and autism, and select six genes labeled as schizophrenia positives that are predicted to be associated with cell motility for follow-up experiments.

**Conclusions:**

Candidate genes predicted by our method suggest testable hypotheses about these genes’ role in cell motility regulation, offering a framework for generating predictions for experimental validation.

**Electronic supplementary material:**

The online version of this article (10.1186/s12859-019-2834-1) contains supplementary material, which is available to authorized users.

## Background

Many *polygenic diseases*, which arise from the action or influence of multiple genes, are difficult to genetically characterize despite strong heritability. For example, schizophrenia and autism are caused by a large number of genetic and environmental variations that perturb numerous processes, but the relationship between the pathophysiology of these diseases and their genetic foundations remains elusive [1, 2]. Genome-wide studies of mutations and gene expression differences have helped characterize the genetic basis of schizophrenia [1, 3–5]. However, extracting causality for symptoms and pathophysiology from these genes remains challenging. Gene expression data reveals minor changes in gene expression levels [3], and the mutations associated with schizophrenia show only slight frequency-of-mutation changes from control participants [4, 5], indicating that schizophrenia arises from a multitude of cellular perturbations compounding their effects.

Schizophrenia and autism have been correlated with aberrations in cell motility, although the mechanisms are unknown [6–10]. Cell motility, the movement of cells through the use of metabolic energy, is vital to numerous cellular processes and is especially important for growth and differentiation of cells. Since axon growth directs neuronal connectivity, regulation of motility may be relevant in neurological diseases. Further, motility assays used in combination with RNAi depletion can be used to validate migration phenotypes [11]. Thus, we aim to find neurological disease genes that may also be involved in cell motility.

Computational methods have been essential to investigate the genetic basis of polygenic disorders. Over the past decade, networks that represent relationships among biological processes have been critical for studying diseases [12]. *Functional interaction networks* integrate vast amounts of genomic and gene interaction data to identify functional similarity between genes [13–15]. These networks can be used to identify which genes are most centrally implicated in a polygenic disease. Given a list of genes that are known or suspected to be altered in a polygenic disease, many methods identify genes that are near the input genes within a functional interaction network [14–20]. These methods use network-based classification approaches such as naïve Bayes [15], clustering [14], support vector machines [18], and Gaussian smoothing [19, 20], which all rely on the connections among genes in the functional interaction network.

We set out to find candidate genes that are associated with cell motility and a polygenic disease such as schizophrenia or autism using functional interaction networks. This application builds upon a classic semi-supervised learning framework for predicting disease genes that labels a small subset of the nodes as positives or negatives and aims to prioritize the remaining unlabeled nodes. Our problem is a phenotype-focused variation on this formulation: we have a polygenic disease (e.g., schizophrenia or autism) and a biological process that is known to be disrupted (e.g., cell motility). Our primary interest is to produce testable hypotheses for aberrant cell motility in candidate schizophrenia or autism genes.

**Contributions**

We formulate the POLYGENIC DISEASE PHENOTYPE Problem, which aims to identify candidate genes associated with a disease that could be experimentally validated by phenotype assays. We develop a Gaussian smoothing method to identify genes that are near known disease genes and cell motility genes in a functional interaction network. Cross validation experiments demonstrate that our ranked candidate genes are more accurate across gold-standard schizophrenia, autism, and cell motility datasets compared to other Gaussian smoothing variants. Further, our method provides a tunable parameter that removes a low-degree bias observed in the highest-ranked candidates of other methods. The top-ranked candidate genes from our method offer a list of potential genes for testing in a cell-based assay of cell motility. Our results provide testable hypotheses for a greater understanding of the relationship between genetic variants associated with schizophrenia and the resulting pathophysiology.

## Methods

A functional interaction network is described as a weighted graph *G*=(*V*,*E*) where the nodes *V* are genes and the undirected edges (*u*,*v*)∈*E* with weights *w*_*uv*_ describe functional similarity between genes. We define a *curated* set of nodes *C*⊂*V* corresponding to genes annotated to a specific disease or biological process. Curated sets may also describe genes not associated with a disease or biological process; we will use $\overline {C} \subset V$ to denote such sets. Curated sets are typically small, since they are usually expensive to collect; thus, the majority of the nodes in *G* are *unlabeled* (they do not appear in either set).

We focus on a specific disease $\mathcal {D}$ (e.g., schizophrenia) and a specific biological process $\mathcal {P}$ (e.g., cell motility). We specify curated sets $C_{\mathcal {D}} \subset V$ and $\overline {C}_{\mathcal {D}} \subset V$ that denote genes associated with $\mathcal {D}$ and not associated with $\mathcal {D}$, respectively, where $C_{\mathcal {D}} \cap \overline {C}_{\mathcal {D}} = \emptyset $. We also specify $C_{\mathcal {P}} \subset V$ that denotes genes associated with $\mathcal {P}$. We wish to solve the following problem:

**POLYGENIC DISEASE PHENOTYPE** (**PDP**) **Problem.** Given a functional interaction network *G*=(*V*,*E*), curated sets $C_{\mathcal {D}}$ and $\overline {C}_{\mathcal {D}}$ for disease $\mathcal {D}$, and a curated set $C_{\mathcal {P}}$ for biological process $\mathcal {P}$, return a prioritized list of candidate genes from *V* predicted to be associated with $\mathcal {D}$ and $\mathcal {P}$ that can be experimentally validated using an assay for $\mathcal {P}$.

As we noted in the Background, our goal is to validate an aberrant biological process in candidate disease genes. Our goal implies an asymmetry to the problem – we are not looking to confirm that genes are involved in a disease; instead we are focusing on the dysregulation of the biological process as a first step towards identifying candidate disease genes associated with the given phenotype.

### Semi-supervised learning methods

There are many network-based classification techniques that incorporate both positive and negative labels. Recently, Krishnan et al. trained an evidence-weighted support vector machine (SVM) classifier to predict candidate autism genes from network features [18]. While this approach was successful, we sought to develop a simpler approach where prioritized genes are closer to positives within a global optimization scheme.

We adapt a Gaussian smoothing method for graphs with positive and negative labels on a subset of the nodes, which aims to find a score for unlabeled nodes that is smooth over the graph topology [21, 22]. This approach has been applied to biological network analysis and implemented as a method called SINKSOURCE, which was originally used to predict HIV dependency factors in a human protein interaction network [20]. SINKSOURCE outperformed six other function prediction algorithms for this task, including methods that use both positive and negative labels and methods that only use positives [20].

Given a curated set *C* of positives and $\overline {C}$ of negatives within a network (the *labeled* nodes $L=C \cup \overline {C}$), we first describe methods to predict labels on the remaining *unlabeled* nodes *U*=*V*∖*L* in *G*. We then introduce a method that incorporates two sets of positives; one from a polygenic disease (e.g. schizophrenia or autism) and one from a biological process (e.g. cell motility).

#### SINKSOURCE [20].

SINKSOURCE models a random Gaussian field on the graph given labeled positive and negative nodes [21, 22]. Let *f*:*V*↦[0,1] be a function where *f*(*v*)=1 if *v*∈*C*, *f*(*v*)=0 if $v \in \overline {C}$, and *f*(*v*) over unlabeled nodes is “smooth” with respect to the topology of *G*. That is, SINKSOURCE chooses values for the unlabeled nodes *U* that minimizes the quadratic equation

1 $$\begin{array}{*{20}l} \min_{f} \frac{1}{2} \sum\limits_{(u,v) \in E} w_{uv} (f(u)-f(v))^{2}, \end{array} $$

conditioned on fixing the values of the labeled nodes *L*. Note that this optimization function is similar to the popular GeneMANIA webserver [19], except that here the labeled nodes have fixed values. The function that minimizes Eq. 1 is harmonic, and so the value of *f* at each unlabeled node is a weighted average of the node’s neighbors [21, 22]:

2 $$\begin{array}{*{20}l} f(v) = \frac{{\sum\nolimits}_{u \in N_{v}} w_{uv} f(u)}{{\sum\nolimits}_{u \in N_{v}} w_{uv}}, \end{array} $$

where *N*_*v*_ is the set of *v*’s neighbors. SINKSOURCE uses an iterative method to calculate *f*(*v*), since it is known to converge [20]. Let *f*_*t*_(*v*) be the value of node *v* at time step *t*; *f*_0_ is initialized as follows:

3 $$\begin{array}{*{20}l} f_{0}(v) = \left\{ \begin{array}{ll} 1 & \text{if } v \in C\\ 0 & \text{if } v \in \overline{C}\\ 0.5 & \text{otherwise.} \end{array}\right. \end{array} $$

For every unlabeled node *v*∈*U*, SINKSOURCE updates *f*_*t*_(*v*) based on the previous timestep:

4 $$\begin{array}{*{20}l} f_{t}(v) = \frac{1}{d_{v}} \sum\limits_{u \in N_{v}} w_{uv} f_{t-1}(u), \end{array} $$

where

$$\begin{array}{*{20}l} d_{v} = \sum\limits_{u \in N_{v}} w_{uv} \end{array} $$

is the weighted degree of node *v*. Equation 4 can be divided into two sums: one sum over the unlabeled nodes *U* and the other sum over the labeled nodes *L*:

5 $$\begin{array}{*{20}l} f_{t}(v) = \underbrace{\frac{1}{d_{v}} \sum\limits_{u \in N_{v} \cap U} w_{uv} f_{t-1}(u)}_{\text{unlabeled neighbor contrib.}} + \underbrace{\frac{1}{d_{v}} \sum\limits_{u \in N_{v} \cap L} w_{uv} f_{t-1}(u)}_{\text{labeled neighbor contrib.}}. \end{array} $$

The function *f*_*t*_(*v*) can be written in matrix form for all unlabeled nodes *U* [20]:

6 $$\begin{array}{*{20}l} \mathbf{f_{t}} = M \mathbf{f_{t-1}} + \mathbf{c}, \end{array} $$

where *f*_*t*_ and *f*_*t*−1_ are vectors of length |*U*|. The contribution from unlabeled neighbors is encoded as a |*U*|×|*U*| matrix *M*:

7 $$\begin{array}{*{20}l} M_{uv} = \frac{w_{uv}}{{\sum\nolimits}_{v \in N_{u}} w_{uv}}. \end{array} $$

The contribution of from the labeled neighbors is encoded as a |*U*|-length vector **c**:

8 $$\begin{array}{*{20}l} c_{v} = \frac{|N_{v} \cap \mathcal{C}|}{{\sum\nolimits}_{v \in N_{u}} w_{uv}}, \end{array} $$

where the numerator is simply the number of positive neighbors (since negative neighbors will contribute a score of 0). The vector **c** can be precomputed because these values are fixed. Since *M* is sparse, calculating *f* using Eq. 6 is more efficient than calculating *f* using Eq. 4. We iteratively compute *f*_*t*_ to calculate *f* for every unlabeled node until either

$$\begin{array}{*{20}l} \sum\limits_{v \in V} \left | f_{t}(v)-f_{t-1}(v) \right | \leq \epsilon \end{array} $$

for some small *ε* (we set *ε*=0.001), or 500 iterations are reached. In practice, the *ε* threshold is reached before 500 iterations in every run of the method. When the method terminates, we have a value *f*(*v*)∈[0,1] for every node in *V*, where unlabeled nodes have a larger score if they are “closer” to labeled positives in *G*.

#### SINKSOURCE+ [20].

Murali et al. also present SINKSOURCE+, a framework that only uses positively-labeled nodes. SINKSOURCE+ introduces a node in the graph *G* that represents a single negative, and connects this node to all |*V*| nodes with a user-defined weight *λ*. Since the introduced node is a negative, its value will never be updated. The labeled nodes *L* are only positives, and the contribution of negatives comes from the single negative node. SINKSOURCE+ incorporates *λ* into the denominator of Eqs. (7) and (8), rather than modifying the underlying graph *G*:

9 $$\begin{array}{*{20}l} M_{uv} = \frac{w_{uv}}{\lambda + {\sum\nolimits}_{v \in N_{u}} w_{uv}}\text{ and } c_{v} = \frac{|N_{v} \cap \mathcal{C}|}{\lambda + {\sum\nolimits}_{v \in N_{u}} w_{uv}}. \end{array} $$

When running SINKSOURCE+, we ignore the labels on negative nodes (we consider them unlabeled).

#### PSEUDO-SINKSOURCE+.

**Fig. 1:**
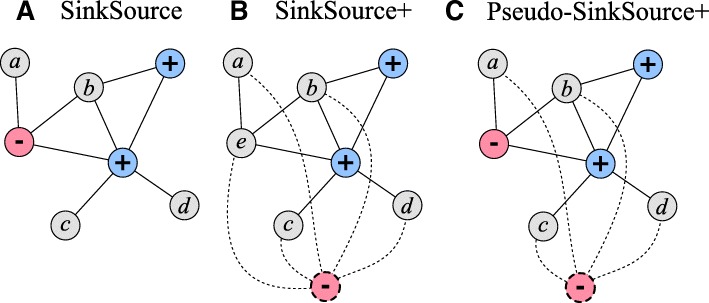
Gaussian smoothing methods for semi-supervised learning. **a**SINKSOURCE uses negative (red) and positive(blue) node labels. **b**SINKSOURCE+ uses only positive labels and introduces a single negative (dashed red node) with *λ*-weighted edges to all unlabeled nodes (dashed edges). **c**PSEUDO-SINKSOURCE+ combines SINKSOURCE and SINKSOURCE+ with a *λ*-weighted sink node and negative node labels

We compared SINKSOURCE and SINKSOURCE+ to a combination of the two methods that includes positives, negatives, and a *λ*-weighted negative node. That is, we use Eq. (9) to add a *λ*-weighted edge from all nodes to an introduced negative node, but we also retain the original negative labels. We call this combination PSEUDO-SINKSOURCE+, where the labeled nodes consist of positives and negatives along with the *λ*-weighted negative node. Figure 1 illustrates PSEUDO-SINKSOURCE+ as a combination of SINKSOURCE and SINKSOURCE+. Note that PSEUDO-SINKSOURCE+ with *λ*=0 is the same as the original SINKSOURCE.

### Solving the PDP problem

We now solve the PDP Problem using the semi-supervised learning formulations described above. Instead of two curated sets *C* and $\overline {C}$, we now have $C_{\mathcal {D}}$ and $\overline {C}_{\mathcal {D}}$ corresponding to disease $\mathcal {D}$ and $C_{\mathcal {P}}$ corresponding to biological process $\mathcal {P}$. Let $learn(G,\lambda,C,\overline {C})$ be the output of a predictive method (SINKSOURCE, SINKSOURCE+, or PSEUDO-SINKSOURCE+) on a graph *G* with *λ*-weighted negative edges and curated sets *C* and $\overline {C}$. We run the method independently for $\mathcal {D}$ and $\mathcal {P}$:

$$\begin{array}{*{20}l} f_{\mathcal{D}} &\gets learn(G,\lambda,C_{\mathcal{D}},\overline{C}_{\mathcal{D}}) \\ f_{\mathcal{P}} &\gets learn(G,\lambda,C_{\mathcal{P}},\overline{C}_{\mathcal{D}}). \end{array} $$

Note that we use the *same* curated set of negatives $\overline {C}_{\mathcal {D}}$, those associated with the disease, because we wish to identify candidates near biological process genes while avoiding genes that are not likely to be associated with the disease.

We finally define a new function *g*:*V*↦[0,1] that combines $f_{\mathcal {D}}(v)$ and $f_{\mathcal {P}}(v)$ for each node *v*∈*V*. There are many forms that *g* can take; here, we let *g* be the product of the two functions:

10 $$\begin{array}{*{20}l} g(v) = f_{\mathcal{D}}(v) f_{\mathcal{P}}(v). \end{array} $$

Our choice of *g* offers the following interpretation for *λ*=0: nodes *v* that are labeled both with the disease $\mathcal {D}$ and the biological process $\mathcal {P}$ (e.g., $C_{\mathcal {D}} \cap C_{\mathcal {P}}$) will automatically have *g*(*v*)=1. Nodes *v* that are labeled with only $\mathcal {D}$ will have $g(v)=f_{\mathcal {P}}(v)$. Conversely, nodes *v* that are labeled with only $\mathcal {P}$ will have $g(v)=f_{\mathcal {D}}(v)$. All other nodes will have some value that is the product of the predicted labels for both $\mathcal {P}$ and $\mathcal {D}$.

### Datasets

#### Functional Interaction Network.

We used a brain-specific functional interaction network from HumanBase [13]. HumanBase, previously called GIANT, catalogs tissue-specific functional interaction networks (http://hb.flatironinstitute.org/). This undirected network was constructed from nearly 1000 genome-scale datasets consisting of both physical interaction and expression measurements, and tissue-specific knowledge was integrated to calculate a posterior probability that each edge connects functionally-related proteins in a tissue [13]. The brain-specific network has been previously used to predict genes associated with autism [18]. The “top edges” network, filtered to include edges with evidence supporting a tissue-specific functional interaction (posterior probability ≥ 0.1), has 333,425,400 edges and 25,825 nodes. Due to the large number of experiments we ran for this paper (over 2000 experiments mostly for cross validation), we removed edges with a posterior probability &lt; 0.15, reducing the network to 3,362,057 edges and 18,095 nodes.

#### Curated Gene Sets.

We compiled two sets of negative genes and three sets of positive genes (two diseases and one biological process) from existing literature.

##### Curated Positive Schizophrenia Genes.

Jia et al. [23] built a curated set of 530 distinct schizophrenia-associated genes based on an integrative analysis of genome-wide association evidence in genetics, epigenetics, transcriptomics, and literature mining [24]. Of these genes, 517 were present in the network and did not overlap with the curated schizophrenia negative set.

##### Curated Negative Schizophrenia Genes.

Of the genes with no significant evidence in SZDB 2.0 [24], we identified those that were not reported to be significantly differentially expressed in other similar polygenic diseases (FDR &gt; 0.2) or schizophrenia itself (FDR &gt; 0.5) [25]. These genes comprised the schizophrenia negative set, and 1561 genes were in the network and did not overlap with the curated positive schizophrenia set or the positive cell motility set.

##### Curated Positive Autism Genes.

Krishnan et al. [18] curated a set of 594 distinct autism-associated genes with evidence ranging from text mining in PubMed abstracts to statistically significant mutations; we considered all genes with evidence as one set of positives. Of these genes, 556 were present in the network and did not overlap with the curated negative set.

##### Curated Negative Autism Genes.

Of the genes that had no evidence in the SFARI autism gene database [26], we identified those that were not reported to be differentially methylated [27] or differentially expressed in other similar polygenic diseases (FDR &gt; 0.2) or autism itself (FDR &gt; 0.7) [25]. These genes comprised the autism negative set, and 973 genes were in the network and did not overlap with the curated positive autism set or the curated positive cell motility set.

##### Curated Cell Motility Genes.

We built a curated set of 542 distinct cell motility-associated genes from the KEGG database [28, 29]. We downloaded genes associated with five key cell motility pathways: the cell adhesion molecule pathway, the focal adhesion kinase pathway, the ErbB signaling pathway, the regulation of actin cytoskeleton pathway, and the tight junction pathway. Of these genes, 526 were present in the network and did not overlap with the curated schizophrenia and autism negative sets.

## Results and Discussion

### Algorithm accuracy and benchmarking

We first evaluated the performance of the Gaussian smoothing approaches on four sets of labeled positive and negative nodes: (1) schizophrenia positives and negatives, (2) cell motility positives with schizophrenia negatives, (3) autism positives and negatives, and (4) cell motility positives with autism negatives. For each case, we have a single set *C* of curated positives and a single set $\overline {C}$ of curated negatives and we rank the nodes by the function *f*.

#### SINKSOURCE and SINKSOURCE+.

**Fig. 2:**
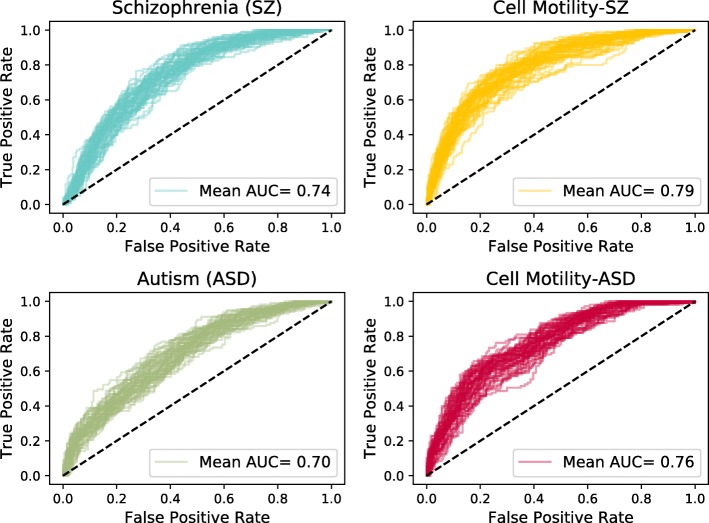
Receiver Operator Characteristic (ROC) curves for five-fold cross validation of SINKSOURCE for schizophrenia, cell motility with schizophrenia negatives, autism, and cell motility with autism negatives. Each plot contains 50 ROC curves

We used five-fold cross validation to assess performance, where we hid one fifth of the positives and one fifth of the negatives from each dataset, ran the smoothing method, and plotted the Receiver Operator Characteristic (ROC) curve using assessing labels of the hidden nodes (Fig. 2). The area under the ROC curve (AUC) values were approximately normally distributed (Additional file 1: Figure S3), and the distribution of AUC values was statistically significant (*p*&lt;0.01) for all pairs of datasets by a two-tailed Welch’s t-test, which tests whether two samples are drawn from normal distributions with the same mean but not necessarily the same standard deviation. Welch’s t-test was used for this and all subsequent statistical tests. The AUC distribution was significantly different for the two datasets with the closest mean AUC (schizophrenia vs. cell motility with autism negatives, *p*=1.60×10^−3^) and the two datasets that have the same set of positives (cell motility) but use different negative sets (schizophrenia negatives vs. autism negatives, *p*=4.99×10^−7^). The cell motility datasets had slightly higher accuracy than the disease datasets; this trend was consistent throughout all analyses. The difference between the cell motility and disease dataset performance is due, in part, to the construction of the curated positives. For example, the cell motility positives had the best accuracy because these were collected from KEGG signaling pathways. These positives tended to be near each other in the functional interaction network since they physically interact with each other.

**Fig. 3:**
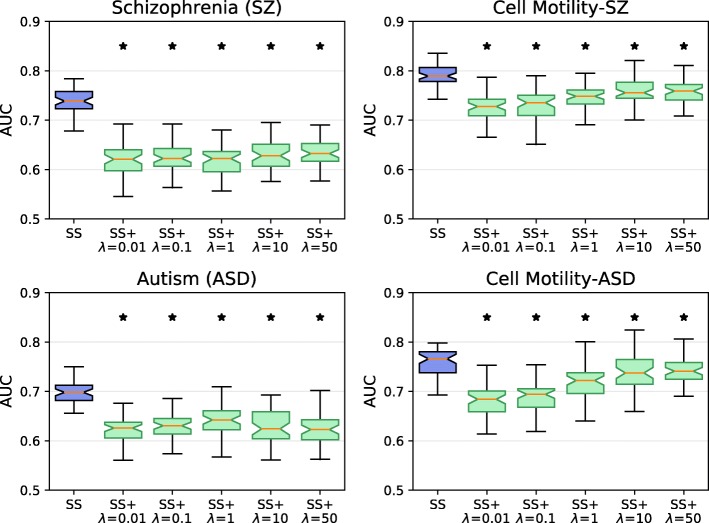
Five-fold cross validation performance (AUC across 50 iterations) of the four datasets predicted using SINKSOURCE (blue) and SINKSOURCE+ (green) for five different values of *λ*. Error bars indicate standard deviation, and asterisks denote significant improvement of SINKSOURCE compared to SINKSOURCE+ (one-tailed Welch’s t-test, *p*&lt;0.01)

We asked whether the curated negative set was important for the method’s accuracy in terms of five-fold cross validation. To assess the impact of the negative set, we first compared SINKSOURCE to SINKSOURCE+ for five values of *λ*-weighted negative edges (Fig. 3), where the SINKSOURCE distribution reflects the ROC curves in Fig. 2. The AUC performance was better when considering negatives for every value of *λ* for all four datasets (one-tailed *p*&lt;0.01), reflecting that the negative set contains useful information to discriminate between positive and negative nodes. In some cases, the performance of SINKSOURCE+ depended on the choice of *λ*. For example, *λ*=50 for the cell motility dataset with autism negatives increased the average AUC by 0.06 compared to *λ*=0.01 (reflecting an 8% increase in accuracy).

**Fig. 4:**
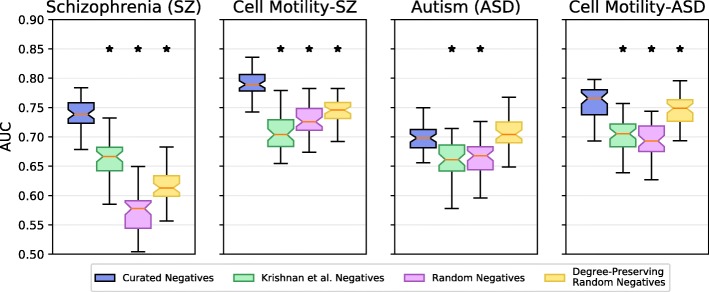
Five-fold cross validation performance of the four datasets predicted using SINKSOURCE with four different negative sets. Number of iterations, error bars, and asterisks are the same as Fig. 3

Next, we compared the accuracy of SINKSOURCE with our curated negative set to other sets of negative nodes (Fig. 4). We considered a set of 1189 distinct genes that are likely associated with non-neurological diseases and were previously used to identify autism-associated genes (the Krishnan et al. dataset) [18]. We emphasize that running SINKSOURCE with the Krishnan et al. positives and negatives cannot be compared to the results from the original publication, since our method does not categorize positives into evidence levels, we do not run the method on a complete graph, and we are not running their SVM. With this in mind, SINKSOURCE with the Krishnan et al. negatives performed worse than SINKSOURCE with our curated set for all datasets (blue vs. green boxplots in Fig. 4). We observed the smallest difference in average AUC performance on the autism dataset, which also utilized the positives gathered from Krishnan et al. However, the AUC distributions were significantly different (*p*=4.8×10^−10^). The schizophrenia dataset had a much larger difference in performance using the Krishnan et al. negatives, suggesting that those negatives may not successfully generalize to other neurological diseases.

We also compared SINKSOURCE with our curated negatives to a random set of negatives from the network and a random set of negatives that preserve the degree distribution of our curated negatives (pink and yellow boxplots in Fig. 4). The random set of negatives performed worse than the curated set in all datasets, and the degree-preserving negatives performed worse than three of the four datasets (one-tailed *p*&lt;0.01). Unsurprisingly, constraining the random negatives by the degree distribution observed in the curated negatives improves performance over random negatives with no degree constraints. In the datasets that use autism negatives, the degree-preserving random negative set performs about as well as the curated set of negatives.

#### Motivation for a new method.

**Fig. 5:**
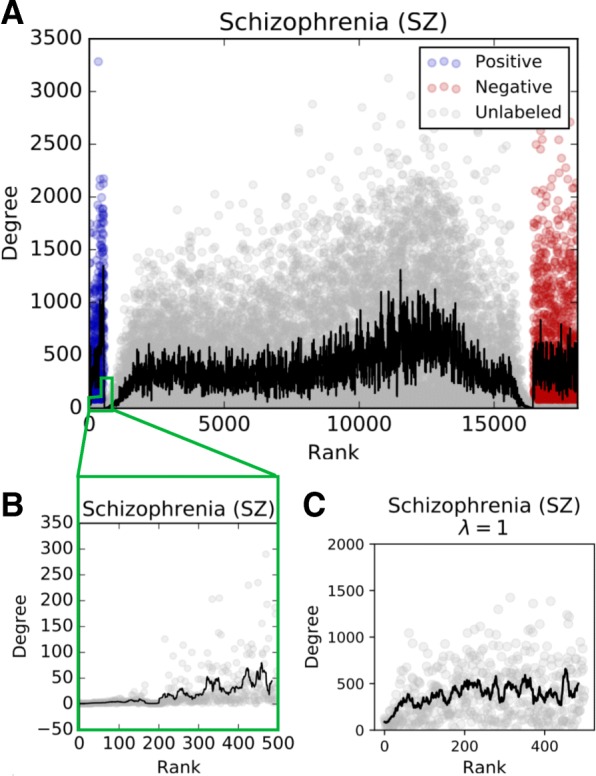
**a** Node ranking (x-axis) by degree (y-axis) of SINKSOURCE run on the schizophrenia dataset. Black line denotes moving average (15 nodes). **b** First 500 unlabeled nodes from **a** illustrating low degree for top-ranked unlabeled nodes. **c** First 500 unlabeled nodes from PSEUDO-SINKSOURCE+ (*λ*=1) run on the schizophrenia dataset

Figures 3 and 4 show that our curated positive and negative sets are reasonable choices for SINKSOURCE in terms of *k*-fold cross validation accuracy. However, when we inspected the top unlabeled nodes ranked by their scores, we found that these genes had very low degree in the network. Figure 5a shows the ranked nodes by degree for the schizophrenia dataset from SINKSOURCE. As expected, the positive nodes were ordered first (blue), then unlabeled nodes (gray), and negative nodes appear last (red). However, the degree of the top-ranked unlabeled nodes for the schizophrenia dataset showed a stark drop compared the labeled positives (Fig. 5b). The other datasets showed a similar trend (first row of Additional file 1: Figure S4). The first 100 unlabeled nodes have an average degree between 2 and 2.6 across the datasets when the average degree of the network is 185. These top-ranked, low-degree unlabeled nodes tended to be connected to positives. If an unlabeled node with degree one was connected to a positive, SINKSOURCE would assign it a final value of 1.0.

#### PSEUDO-SINKSOURCE+.

Based on this observation, we wanted to adjust the predictions for these low-degree nodes that are skewed based on their neighbors. There are many ways to make this adjustment. For example, GeneMANIA predicts the values of labeled nodes instead of keeping the labeled scores fixed [19]. However, GeneMANIA will still have the issue that a degree-one node connected to a positive will always be given that positive’s score. PSEUDO-SINKSOURCE+ considers negatives (as SINKSOURCE does) and makes uses of a *λ*-weighted negative edge (as SINKSOURCE+ does).

We found that SINKSOURCE+ helped correct the high-ranking, low-degree node issue with large values of *λ*. Specifically, very few of the unlabeled nodes of the top 500 candidates had low degree for PSEUDO-SINKSOURCE+ with *λ*=1 (Fig. 5c). The average degree of the first 100 unlabeled nodes increased to between 198 and 229 across the four datasets, compared to about 2 for SINKSOURCE. This trend was consistent with different values of *λ* (Additional file 1: Figure S4).

**Fig. 6:**
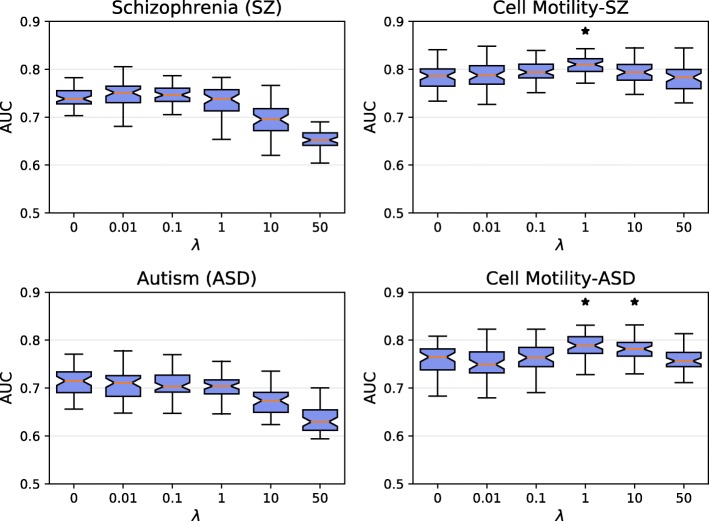
Five-fold cross validation performance of PSEUDO-SINKSOURCE+ for the four datasets across six values of *λ*. Number of iterations, error bars, and asterisks are the same as Fig. 3

We next evaluated the accuracy of PSEUDO-SINKSOURCE+ as *λ* increases (Fig. 6). In the schizophrenia and autism datasets, the accuracy remained about the same for small values of *λ* and deteriorated for *λ*≥10. However, in the cell motility datasets, accuracy held steady with increasing values of *λ*, even significantly outperforming the *λ*=0 (original SINKSOURCE) case (asterisks in Fig. 6). As we increased *λ*, PSEUDO-SINKSOURCE+ with the curated negatives usually outperformed the method with other negative sets (Additional file 1: Figure S5). These results indicate that *λ* can be tuned to allow higher-degree nodes to be ranked earlier without sacrificing accuracy in terms of AUC values. For the remaining results we used PSEUDO-SINKSOURCE+ with *λ*=1, as this value did not deteriorate accuracy for the disease datasets and improved accuracy for the cell motility datasets.

#### Multi-layer PSEUDO-SINKSOURCE+

PSEUDO-SINKSOURCE+ corrected the low-degree bias by varying a *λ*-weighted negative contribution to all nodes. We also sought to develop a method where a node’s value of *f* included contributions from more distantly labeled nodes whose effect were drowned out by adjacent positively-labeled nodes. For example, in Fig. 1a, the value of node *c* could be a weighted combination of the existing graph with the immediately labeled neighbor (contributing a score of 1) and the graph where the neighbor’s label is hidden, allowing the other labeled nodes to influence *c*’s score. Our approach makes *l* copies of the original graph *G* and partitions the labeled nodes across these *l* “layers.” The number of layers *l* is a user-defined parameter. The copies of node *v* (e.g., *v*_1_,*v*_2_,…,*v*_*l*_) are connected to a supernode *v*_0_ introduced for each node from the original graph *G*. We also include the *λ*-weighted negative edges as in PSEUDO-SINKSOURCE+. The same Gaussian smoothing process determines different values *f*(*v*_1_),*f*(*v*_2_),…,*f*(*v*_*l*_) for each copied node. The final value of node *v* is calculated as *f*(*v*_0_), the weighted average of *v*’s copies in the modified graph. More details about the multi-layer approach, including a motivating example, are provided in Additional file 1: Section S1.

We ran MULTI-LAYER PSEUDO-SINKSOURCE+ for *l*=2 and *l*=3 layers and compared the results to PSEUDO-SINKSOURCE+. When *λ*=0, the top nodes ranked by the multi-layer method have an even worse degree bias than SINKSOURCE. However, as *λ* increases, additional layers helps correct the degree bias more than PSEUDO-SINKSOURCE+ (Additional file 1: Figure S6). Despite this further correction, partitioning the labeled nodes across layers does not improve the cross validation accuracy compared to PSEUDO-SINKSOURCE+ (Additional file 1: Figure S7). Further, the size of the modified graph with multiple layers increases the running time of the Gaussian smoothing method. For these reasons, we returned to PSEUDO-SINKSOURCE+ because it corrects the low-degree bias in a simple manner while retaining accuracy for reasonable values of *λ*.

### Schizophrenia and cell motility predictions

We used PSEUDO-SINKSOURCE+ with *λ*=1 to solve the PDP Problem for schizophrenia and cell motility. We used the schizophrenia positives $C_{\mathcal {D}}$ and the cell motility positives $C_{\mathcal {P}}$ and calculated three ranked nodes: predicted schizophrenia candidates $f_{\mathcal {D}}$, predicted cell motility candidates $f_{\mathcal {P}}$, and the combined score *g* (Eq. (10)). The **f** vectors were normalized by the largest score for a node in *V*_0_, so they were in the range of [0,1]. The combined score *g* was not normalized, but rather a product of $f_{\mathcal {D}}$ and $f_{\mathcal {P}}$.

#### Comparison to the union of the positive sets.

We asked whether running PSEUDO-SINKSOURCE+ with the union of the schizophrenia and cell motility positives would produce a better ranking than running the two positive sets separately and combining them into one final score *g*. Thus, we calculated the additional predictor $f_{\mathcal {D} \cup \mathcal {P}}$ for *λ*=1:

11 $$\begin{array}{*{20}l} f_{\mathcal{D} \cup \mathcal{P}} & \gets learn(G,\lambda,C_{\mathcal{D}} \cup C_{\mathcal{P}},\overline{C}_{\mathcal{D}}), \end{array} $$

which was normalized by the largest value of *V*_0_.

**Fig. 7:**
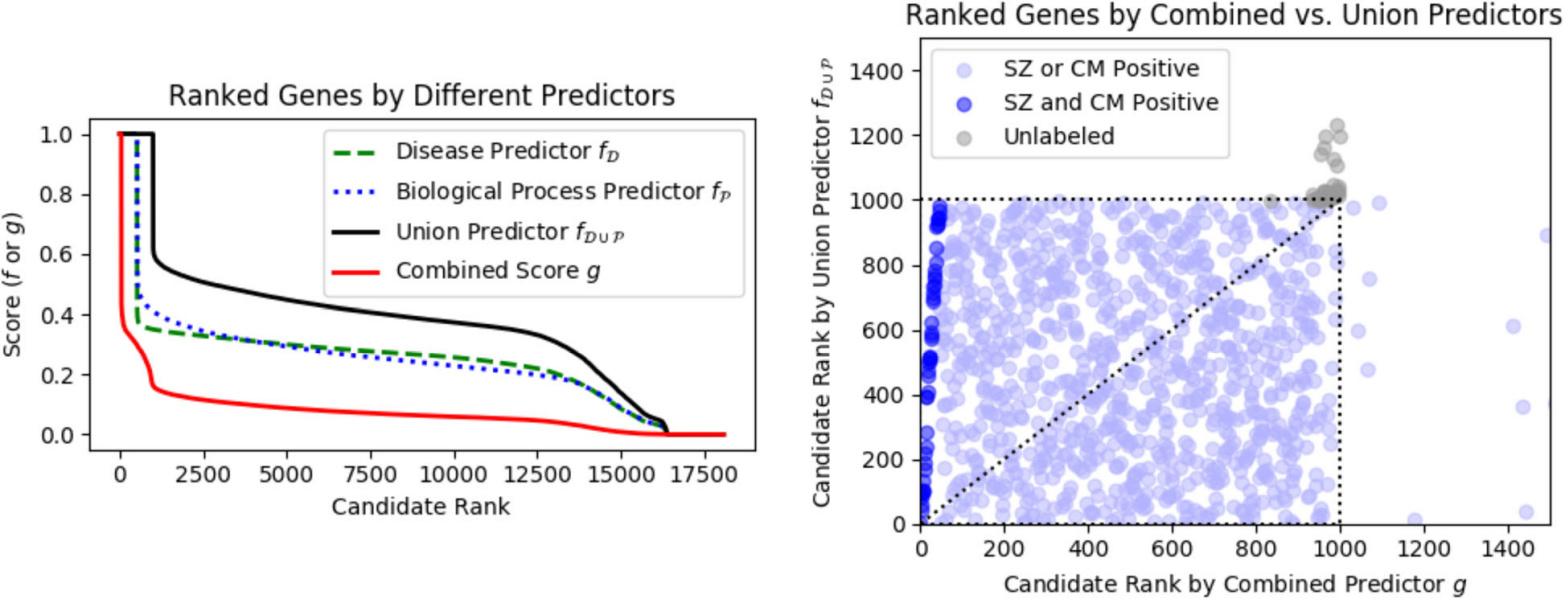
PSEUDO-SINKSOURCE+ runs on different positive sets for *λ*=1 for schizophrenia as the disease set $\mathcal {D}$. (Left) Genes ranked by score according to schizophrenia $f_{\mathcal {D}}$, cell motility $f_{\mathcal {P}}$, union $f_{\mathcal {D} \cup \mathcal {P}}$ and combined score *g*. (Right) Scatter plot of gene rankings in combined *g* vs. union $f_{\mathcal {D} \cup \mathcal {P}}$. Each point is a gene, and the first 1000 ranked nodes in each method are plotted (dotted box)

The predicted scores for these four experiments followed relatively similar distributions across all ranked nodes (Fig. 7 left). The predictor *g* produced the smallest scores, which was reasonable because it was the product of two of the other predictors. The union predictor produced many nodes with large scores due to the fact that the number of positives nearly doubled in this setting. The top-ranked candidates between the combined predictor *g* and the union predictor $f_{\mathcal {D} \cup \mathcal {P}}$ were notably different (Fig. 7 right). In this figure, if the two methods had generated identical rankings, we would see a diagonal line. Nodes that were labeled as both schizophrenia and cell motility positives appeared at the very top of predictor *g*’s ranking compared to predictor $f_{\mathcal {D} \cup \mathcal {P}}$ (dark blue points). In the combined score method, these nodes had large $f_{\mathcal {D}}$ and $f_{\mathcal {P}}$ scores, which distinguished them from nodes that have only one large score. Note that genes ranked in the top 1000 for one method may not have appeared in the top 1000 for the other (points outside the dotted box); see Additional file 1: Figure S8 for the full range of values. As expected, the union method promoted the 997 genes that were labeled as a positive in either set before ranking a node that was unlabeled in either set. In comparison, nodes that were unlabeled in either set were ranked between 900 and 1000 in the combined method but ranked worse than 1000 in the union method (the group of gray points). Thus, the combined method has the potential to promote nodes that are unlabeled in either set over the nodes that are positive in exactly one of the sets.

#### Predicted Candidates.

From Fig. 7, it is clear that many of the top-ranked candidates for the combined score will either be a schizophrenia positive, cell motility positive, or both. The cases where the node is a positive in both sets is uninteresting from a predictive sense. Instead, we focused on the candidates in the top 70 predictions (sorted by combined score *g*) that were unlabeled (bold) in one or both of the positive sets (Table 1). Note that all these candidates would be tied using the union method ($f_{\mathcal {D} \cup \mathcal {P}}$ column). The candidates in the table were labeled as positive in one of the sets; the first node that was unlabeled in both sets is CD74 at rank 972. Our approach generalizes beyond schizophrenia data as well; we performed the same analysis with the autism dataset and report the top-ranking candidate genes in Additional file 1: Table S1.

**Table 1:** Candidate genes associated with schizophrenia ($\mathcal {D}$) and cell motility ($\mathcal {P}$), ordered by their combined score *g*(*v*)

Gene Name	Entrez	Rank	Deg	$f_{\mathcal {D}}$	$f_{\mathcal {P}}$	$f_{\mathcal {D}\cup \mathcal {P}}$	*g*(*v*)
EMILIN1	11117	47	113	*1.00*	**0.46**	1.00	0.46
IFITM3	10410	48	386	*1.00*	**0.45**	1.00	0.45
TGM2	7052	49	420	*1.00*	**0.44**	1.00	0.44
SEMA3A	10371	50	173	*1.00*	**0.44**	1.00	0.44
CLU	1191	51	549	*1.00*	**0.44**	1.00	0.44
ADAMTS3	9508	52	113	*1.00*	**0.43**	1.00	0.43
AIF1	199	53	223	*1.00*	**0.43**	1.00	0.43
LRP4	4038	54	184	*1.00*	**0.43**	1.00	0.43
RPTOR	57521	55	8	*1.00*	**0.42**	1.00	0.42
HLA-DRB5	3127	56	93	**0.42**	*1.00*	1.00	0.42
PMP22	5376	57	558	*1.00*	**0.42**	1.00	0.42
DAB2	1601	58	624	*1.00*	**0.42**	1.00	0.42
PAK3	5063	59	114	**0.42**	*1.00*	1.00	0.42
EGR1	1958	60	317	*1.00*	**0.42**	1.00	0.42
CDH13	1012	61	37	*1.00*	**0.42**	1.00	0.42
PTPRG	5793	62	325	*1.00*	**0.42**	1.00	0.42
GRK5	2869	63	546	*1.00*	**0.41**	1.00	0.41
PTGS2	5743	64	202	*1.00*	**0.41**	1.00	0.41
SYT11	23208	65	273	*1.00*	**0.41**	1.00	0.41
TNFAIP2	7127	66	479	*1.00*	**0.41**	1.00	0.41
GPM6B	2824	67	565	*1.00*	**0.41**	1.00	0.41
GALNT10	55568	68	322	*1.00*	**0.40**	1.00	0.40
LRP1	4035	69	877	*1.00*	**0.40**	1.00	0.40
HES1	3280	70	147	*1.00*	**0.40**	1.00	0.40

Genes in the top 70 ranking that are unlabeled in either in $\mathcal {D}$ and $\mathcal {P}$ are shown. Italic font indicates that the gene is positively labeled; bold font indicates that the gene is unlabeled

**Fig. 8:**
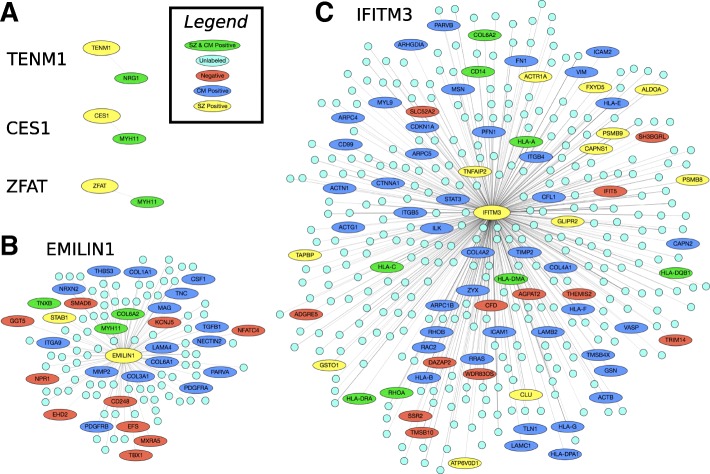
Representative networks showing top candidates and their neighbors for the combined method on the schizophrenia dataset. **a** The three nodes tied for the top ranking according to SINKSOURCE (TENM1, CES1, and ZFAT). **b** The top-ranked node and **c** the second-best node according to PSEUDO-SINKSOURCE+ with *λ*=1

To illustrate the dramatic difference in network topology for the candidates determined by SINKSOURCE compared to PSEUDO-SINKSOURCE+, we visualized the neighbors of the top-ranked nodes from the two methods for schizophrenia using GraphSpace, an interactive web-based visualization server [30]. Three nodes were tied for the best-ranked candidates in SINKSOURCE that were unlabeled in at least one of the curated positive sets (schizophrenia or cell motility) – these three nodes had a score of 1.0 and a single neighbor that happened to be a positive in both sets (Fig. 8a). The two top-ranked nodes determined by PSEUDO-SINKSOURCE+ with *λ*=1, on the other hand, had 113 neighbors and 386 neighbors, respectively, many of which were unlabeled (cyan nodes in Fig. 8b and c).

Our overall goal is to identify candidate genes that are associated with schizophrenia and may exhibit an altered cell motility phenotype, and we have an RNAi assay in *Drosophila* cells to test the effect of a candidate gene on cell movement. We further examined the list in Table 1 for candidates that were (a) supported in the literature as being involved in motility [31, 32], (b) highly conserved in flies by a BLAST sequence alignment [33], (c) highly expressed in *Drosophila* D25c cells [32], and (c) were not involved in a large number of biological processes. These aggressive filters removed many of the top candidates. For example, consider the first three nodes in Table 1. EMILIN1 is involved in the development of elastic tissues, but it is not highly conserved in flies; IFITM3 is an immunity protein associated with the flu so it is not directly relevant; and TGM2 interacts with integrins and other adhesion proteins, but it is poorly expressed in D25c cells [31, 32]. Working through the list of candidates, we selected six candidates for follow-up investigation, including AIF1, PTPRG, and GRK5 (ranked 53, 62 and 63 in Table 1, respectively). AIF1, known as Swiprosin-1 in flies, is an actin-binding protein that plays a role in Rac signaling [31]. PTPRG, known as Ptp99A in flies, is a phosphatase that alters motor axon phenotypes and is associated with motor axon defects [32]. GRK5, known as GPRK2 in flies, is a kinase that has been reported to be differentially methylated in schizophrenia studies [23, 32]. Other candidates are SNAP91 (like-AP180 in flies, ranked 107), CLTCL1 (clathrin heavy chain isoform A in flies, ranked 353) and CNTN4 (LD28757p in flies, ranked 720).

## Conclusion

We introduced the POLYGENIC DISEASE PHENOTYPE Problem to predict disease genes that may be associated with a phenotype of interest using a functional interaction network. In this work, we focused on schizophrenia and autism and investigated genes that may be associated with changes in cell motility patterns, a phenotype that has been observed in both diseases. We first demonstrated that our curated positives and negatives perform well in terms of cross-validation accuracy for SINKSOURCE, a Gaussian smoothing method. However, the top-ranked nodes from this approach had very low degree, in part due to the fact that at least one of their few neighbors was a positive. This effect placed more emphasis on nodes with low degree and a few positives, compared to more connected nodes with a larger number of positive neighbors. We then showed that PSEUDO-SINKSOURCE+, a combination of previous Gaussian smoothing methods, corrected this low-degree bias while retaining comparable cross validation accuracy (and, in some settings, improved the accuracy).

In PSEUDO-SINKSOURCE+, the choice of *λ* uniformly dampens the predictions by increasing the denominator of the score function, while the set of negatives selectively dampens the predictions for neighbors of negatives. An outstanding question is how to select *λ* properly. In our experiments, *λ*=1 produced the most accurate candidates; however, it is unclear how to set *λ* when the limited amount of labeled data prohibits cross-validation. One way to select *λ* is to find a value where the top-ranked candidates from PSEUDO-SINKSOURCE+ have an average degree similar to what is observed in the full network (e.g. about 176 neighbors on average), but this would suggest a single *λ* that is independent of the disease or process. Different values of *λ* may be better for different datasets; if there are enough positively-labeled nodes, one can compare the average degree of the positives (e.g. about 535 neighbors for schizophrenia) with the average degree of the same number of top-ranked candidates. However, the degree distribution of the top unlabeled nodes is notably different from the degree distribution of the positive set across all four experiments, indicating that this approach is likely overly simplistic (Additional file 1: Figure S9). Compiling a list from multiple runs of PSEUDO-SINKSOURCE+ with different choices of *λ* values may provide a more comprehensive prediction of functional association. Another consequence of our method is that unlabeled nodes will never be ranked higher than the labeled positives when considering a single curated set of positives and negatives; other methods such as GeneMANIA [19] relax this assumption. Ongoing work includes automatically determining a proper choice of *λ*, modifying the Gaussian smoothing method to predict top nodes that reflect the curated positive degree distribution, and exploring other semi-supervised learning methods for this problem.

Based on our results, we have selected six candidates to experimentally test their effect on cell motility in a cell-based assay. This selection was based on additional post-processing steps, and ultimately done with manual checks. Automating this type of downstream analysis will accelerate the selection of candidates for experimental screening. Overall, our work presents a framework for investigating biological processes that may be disrupted in polygenic diseases. The problem formulation and computational approach opens many directions of further research that leverages computational knowledge to inform experiments for complex disease phenotypes.

## Additional file

Additional file 1 Details about Multi-Layer Pseudo-SinkSource+, supplementary figures, and one supplementary table. (PDF 1010 kb)

## Abbreviations

ASD: Autism spectrum disorder
AUC: Area under the ROC curve
FDR: False discovery rate
PDP: Polygenic Disease Phenotype
ROC: Receiver operating characteristic
SS: SinkSource
SVM: Support vector machine
SZ: Schizophrenia
