# Supplementary material for: Network-based prediction of polygenic disease genes involved in cell motility
Source: BMC Bioinformatics. 2019 Jun 20;20(Suppl 12):313. doi: 10.1186/s12859-019-2834-1 (PMC6584515; doi:10.1186/s12859-019-2834-1)
Supplement: Supplementary file 1 — Details about Multi-Layer Pseudo-SinkSource+, supplementary figures, and one supplementary table. (PDF 1010 kb) [file 12859_2019_2834_MOESM1_ESM.pdf]

Supplementary Information for

Network-Based Prediction of Polygenic Disease Genes Involved in Cell Motility

Miriam Bern*[1], Alexander King*[1], Derek Applewhite[1] and Anna Ritz†[1]

[1]Biology Department, Reed College, Portland, OR

All software and datasets are publicly available and provided on the supplementary website:

`https://github.com/annaritz/CREU-szgene-predictor`.

Contents

S1 Supplementary Methods: Multi-Layer Pseudo-SinkSource+ **2**

S2 Supplementary Figures **4**

S3 Supplementary Tables **11**

*These authors contributed equally to this work.

†aritz@reed.edu

S1 Supplementary Methods: Multi-Layer Pseudo-SinkSource+

We propose a modification to the input graph G that promotes genes near many positives over low-degree genes that are near only one or two positives. Given the undirected, weighted interactome G, curated sets C and $\overline{C}$, and a small integer k[1], we construct a new graph $G' = (V', E')$ as follows (Figure S1):

1. Make k copies of G to produce
$$G_1 = (V_1, E_1),\ G_2 = (V_2, E_2),\ \ldots,\ G_k = (V_k, E_k).$$
Edges retain their weights from the original graph G.

2. Make one additional copy V_0 of the node set V. There are now $k+1$ copies of each node (e.g., $v_0, v_1, \ldots, v_k$).

3. Introduce edges that connect each node in V_0 to its copies in each of the other graphs. That is, we define edges
$$(v_0, v_1), (v_0, v_2), \ldots, (v_0, v_k)$$
for all nodes $v_0 \in V_0$. Each node in V_0 will then have k edges. Let these edges be defined by E_0; each edge in E_0 is given a user-defined weight (we use a constant weight of 1.0).

The new graph $G' = (V', E')$ is the union of the nodes and edges from these copies:

$$V' = \bigcup_{i=0}^{k} V_i \text{ and } E' = \bigcup_{i=0}^{k} E_i. \tag{1}$$

The labeled negatives and positives are randomly partitioned into k groups, which are then added to the graph copies G_1 through G_k (Figure S1(B)).

Finally, we introduce the single sink node, connected to all nodes in V' with weight λ. We test λ values across three orders of magnitude: 0, 0.01, 0.1, 10, and 50. We call this method Multi-Layer Pseudo-SinkSource+; note that One-Layer Pseudo-SinkSource+ with $\lambda = 0$ corresponds to the original SinkSource method, and One-Layer Pseudo-SinkSource+ with $\lambda > 0$ corresponds to Pseudo-SinkSource+.

[1]In the main manuscript, this parameter is called l.

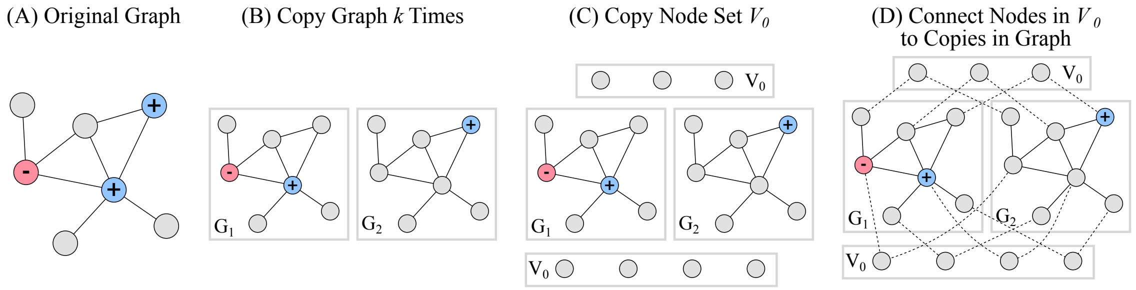

Figure S1: Multi-Layer Graph Construction for $k = 2$ layers and $\lambda = 0$. (A) Original (labeled) graph G, and (B–D) graph G' built from G. Positives and negatives are partitioned across the graph copies in (B). In Panel (D), dashed edges denote E_0; λ-weighted sink node not shown.

We take the predicted labels from $V_0 \in V'$ as the final node scores, which combines the predictions from each of the k networks using a weighted average based on the user-defined edge weights for E_0 (Figure S1(B–D)). Note that the nodes V_0 ensure that the predicted values for $v_1, v_2, \ldots, v_k$ are similar due to the Gaussian smoothing function.

Depending on the value of λ, the predicted labels from V_0 may be very low. For example, if $\lambda = 1$ with $k = 2$ layers, then $\frac{1}{3}$ of the contribution for every $v \in V_0$ will be from a labeled negative. Thus, as a post-processing step, we normalize the predicted labels in V_0 to be between 0 and 1 by dividing by the maximum predicted value from V_0.

Figure S2 illustrates the difference between Multi-Layer Pseudo-SinkSource+ with one and two layers, where nodes c and d will automatically be assigned a value of 1 in the one-layer example.

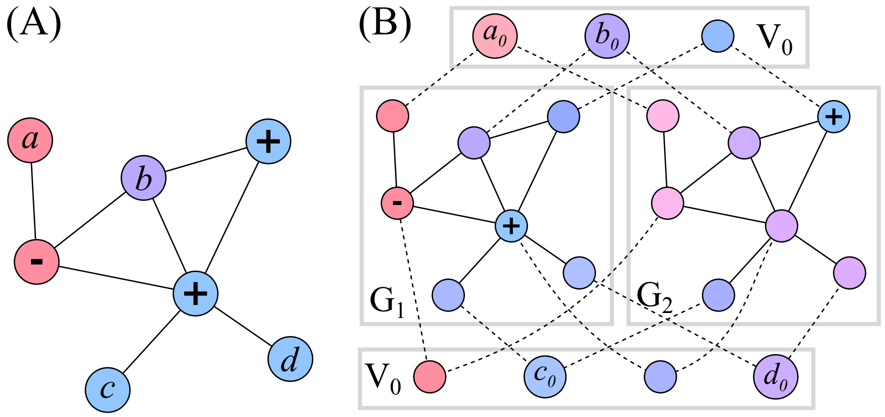

Figure S2: Two-Layer Pseudo-SinkSource+ example with $\lambda = 0$. Nodes are colored according to proximity to labeled positives and negatives. (A) In the one-layer method, c and d will both be assigned a score of 1. (B) In the two-layer method, c and d will be assigned different scores due to the contributions from two layers.

S2 Supplementary Figures

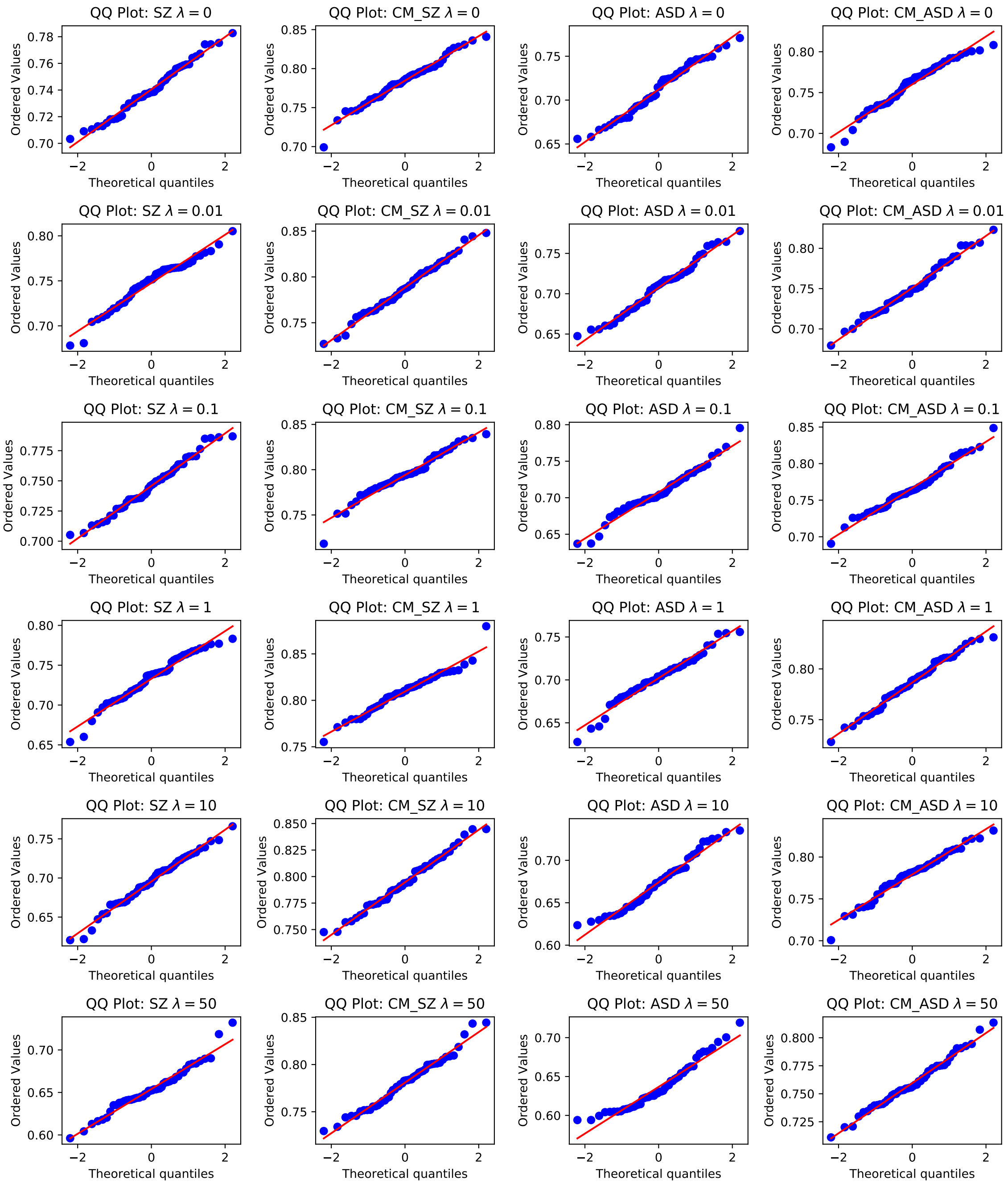

Figure S3: Gaussian Quantile-Quantile plots for the distribution of AUC values in k-fold cross validation runs for Pseudo-SinkSource+. Columns denote the experiment: schizophrenia (SZ), cell motility with SZ negatives (CM_SZ), autism (ASD), and cell motility with ASD negatives (CM_ASD). Rows denote the λ value (0,0.01,0.1,1,10, and 50). Note that Pseudo-SinkSource+ with $\lambda = 0$ is the same as SinkSource.

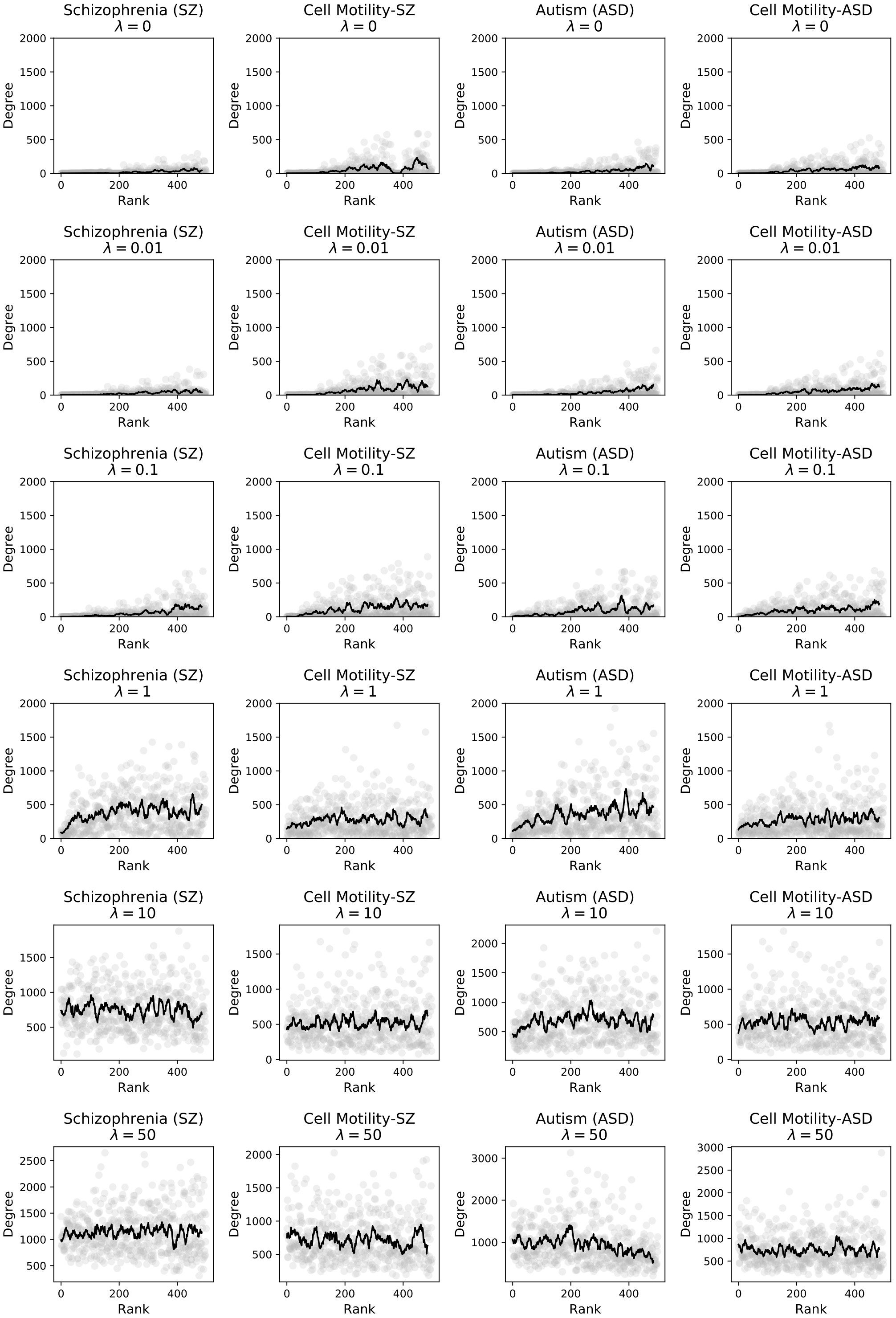

Figure S4: Node ranking (x-axis) by degree (y-axis) for the first 500 unlabeled nodes for PSEUDO-SINKSOURCE+ run on each dataset. Black line denotes moving average (15 nodes).

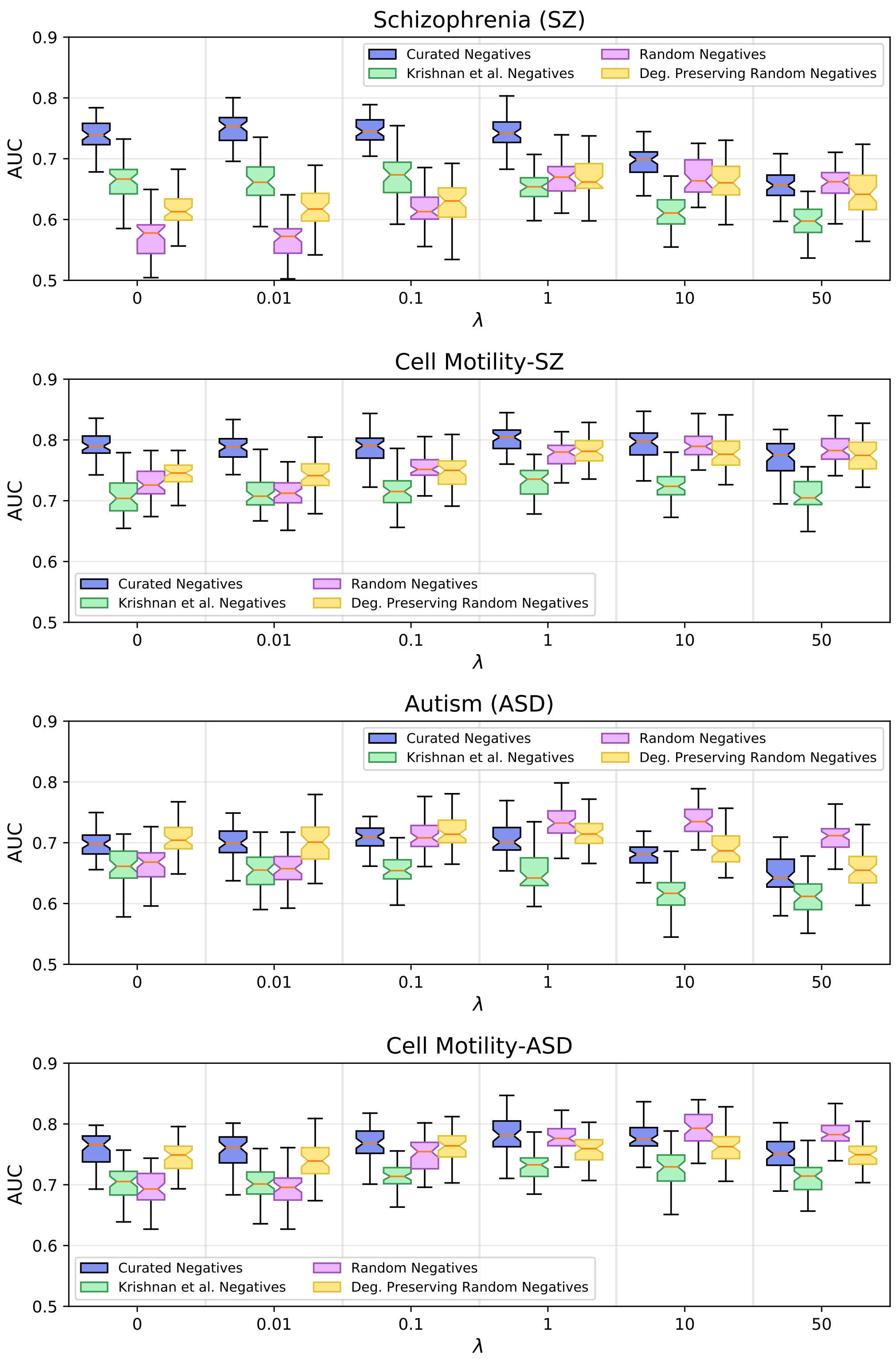

Figure S5: Five-fold cross validation performance (AUC across 50 iterations) of PSEUDO-SINKSOURCE+ with the curated negatives (blue) compared to the method run with the Krishnan et al. negatives (green), random negatives (pink), and random negatives preserving the degree distribution of the curated negative set (yellow).

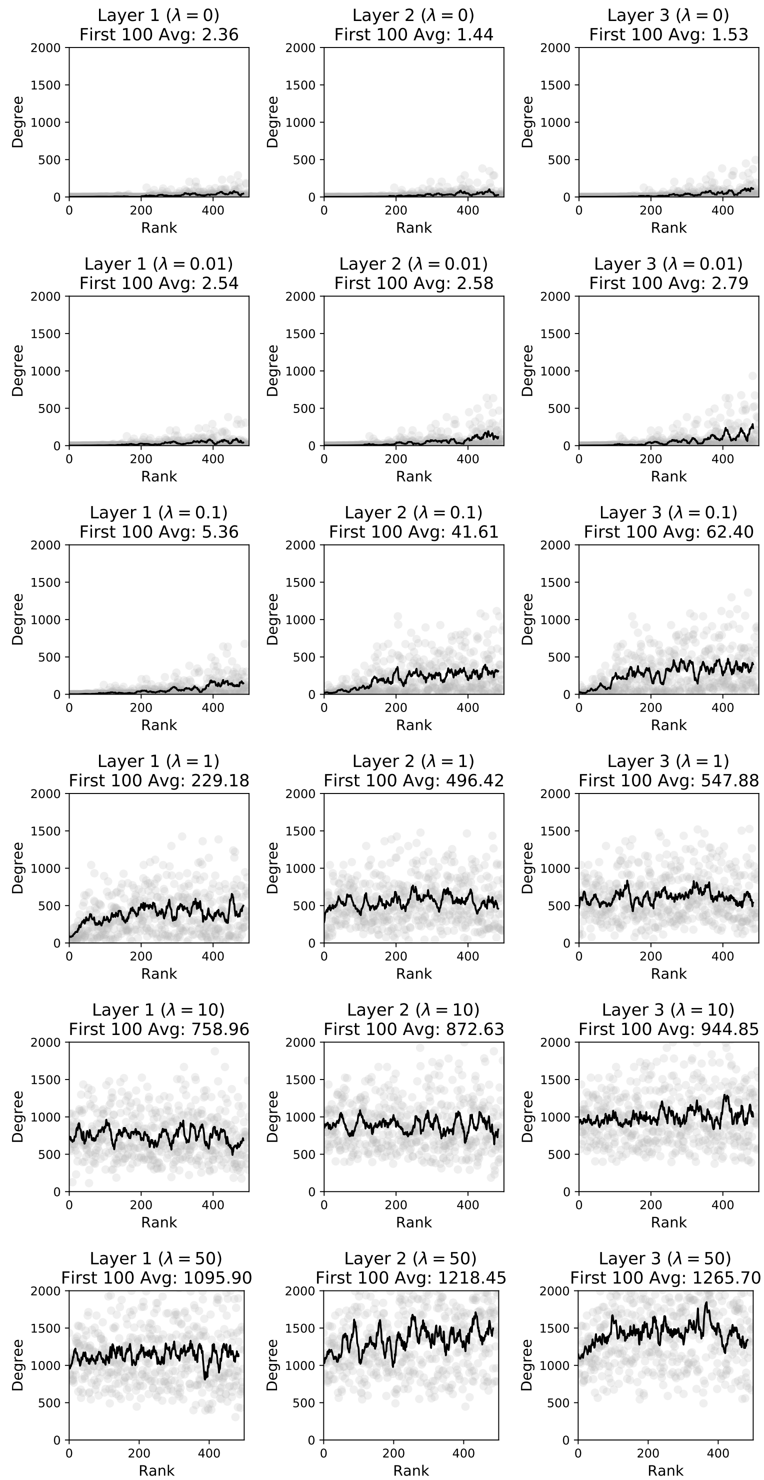

Figure S6: Node ranking (x-axis) by degree (y-axis) for the first 500 unlabeled nodes for Multi-Layer Pseudo-SinkSource+ with one (left), two (middle), or three (right) layers on the schizophrenia dataset. Black line denotes moving average (15 nodes). Note that One-Layer Pseudo-SinkSource+ is equivalent Pseudo-SinkSource+, and that One-Layer Pseudo-SinkSource+ with $\lambda = 0$ is equivalent to SinkSource.

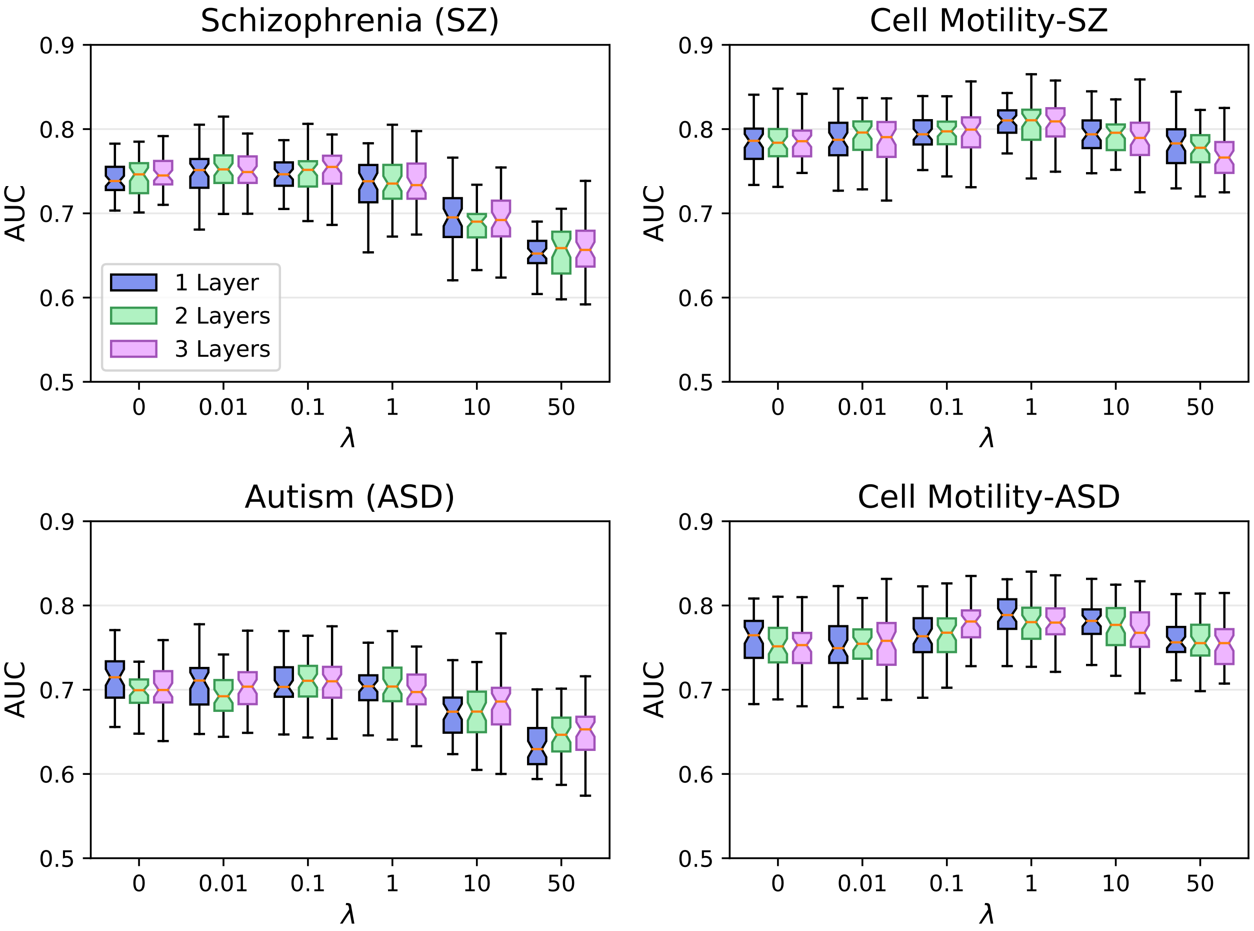

Figure S7: Five-fold cross validation performance (AUC across 50 iterations) of Multi-Layer Pseudo-SinkSource+ for one layer (Pseudo-SinkSource+, blue), two layers (green), and three layers (pink) across different values of λ. Details about the Multi-Layer Pseudo-SinkSource+ method can be found in the Supplementary Methods.

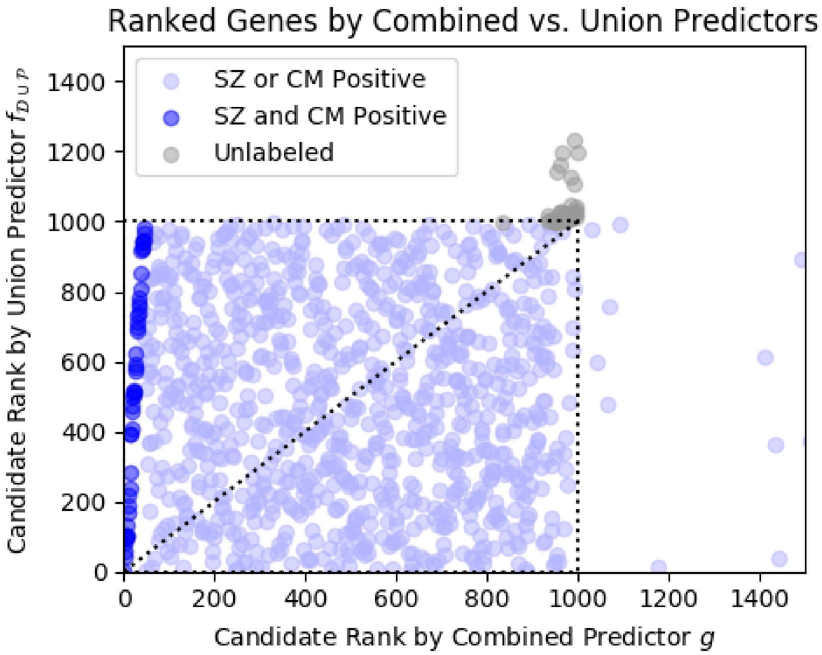

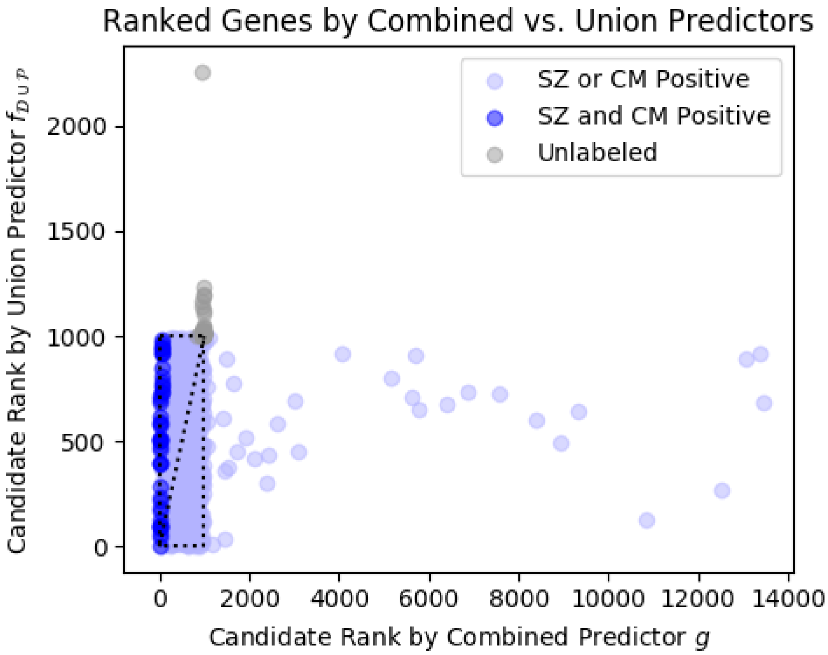

Figure S8: (Top, Figure 7 right) Scatter plot of gene rankings in combined g vs. union $f_{\mathcal{D}\cup\mathcal{P}}$. Each point is a gene, and the first 1,000 ranked nodes in each method is plotted (dotted box). (Bottom) Full version top figure.

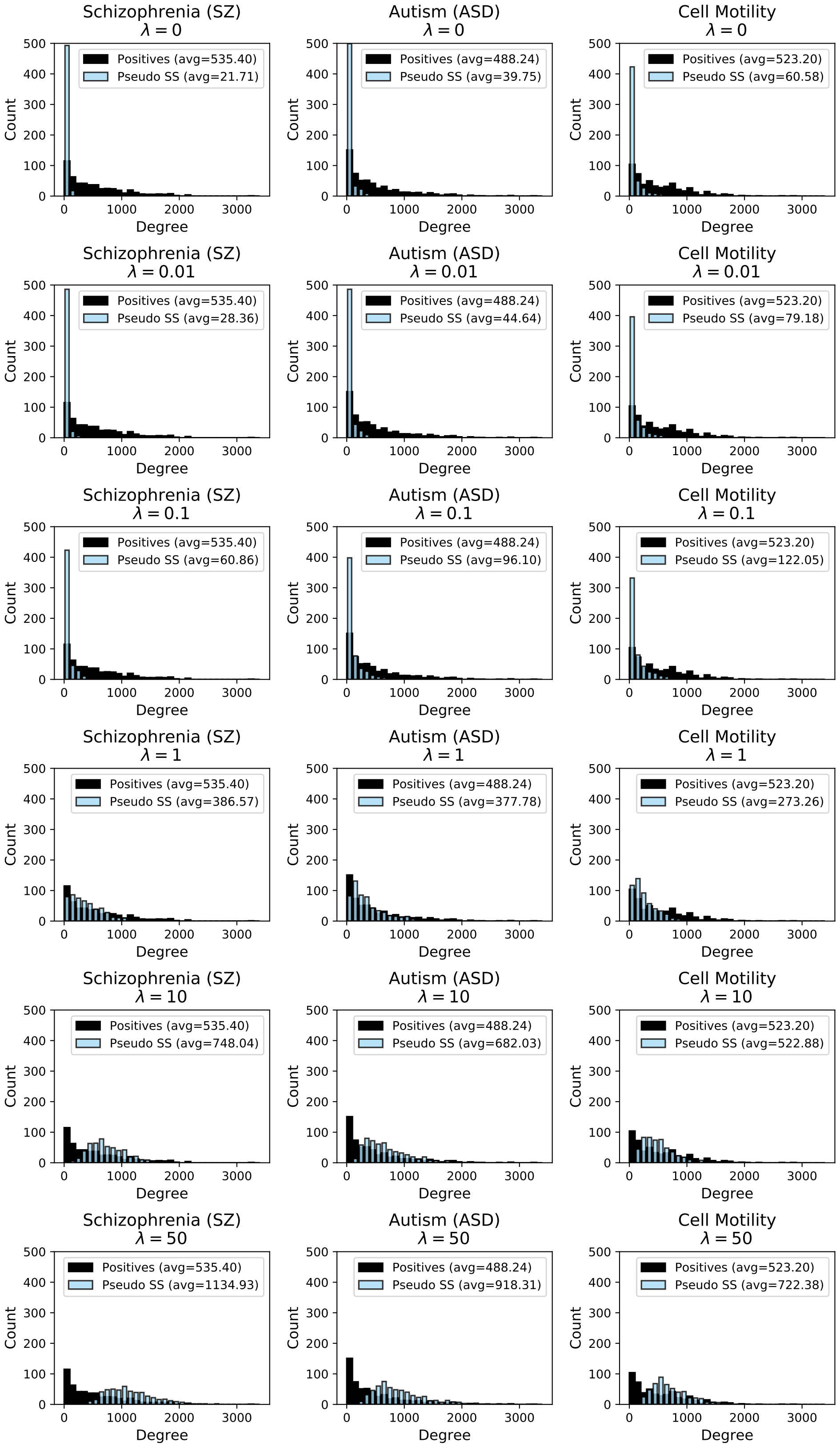

Figure S9: Degree distribution of curated positives (black) and the same number of the top-ranked unlabeled nodes from Pseudo-SinkSource+ (blue) for different values of λ.

S3 Supplementary Tables

Gene Name	Entrez	Rank	Deg	$f_{\mathcal{D}}$	$f_{\mathcal{P}}$	$f_{\mathcal{D}\cup\mathcal{P}}$	$g(v)$
SHANK3	85358	51	64	*1.00*	**0.56**	1.00	0.56
PAK3	5063	52	114	**0.55**	*1.00*	1.00	0.55
CTNNA2	1496	53	23	**0.55**	*1.00*	1.00	0.55
NCAM2	4685	54	107	**0.55**	*1.00*	1.00	0.55
SHC3	53358	55	258	**0.54**	*1.00*	1.00	0.54
PAK5	57144	56	217	**0.54**	*1.00*	1.00	0.54
CNTN1	1272	57	428	**0.51**	*1.00*	1.00	0.51
CAMK2A	815	58	1081	**0.51**	*1.00*	1.00	0.51
CHRM2	1129	59	101	**0.51**	*1.00*	1.00	0.51
CNTN2	6900	60	692	**0.51**	*1.00*	1.00	0.51
NRXN3	9369	61	815	**0.51**	*1.00*	1.00	0.51
RASGRF1	5923	62	1025	**0.51**	*1.00*	1.00	0.51
PPP2R2B	5521	63	788	**0.51**	*1.00*	1.00	0.51
CADM3	57863	64	1009	**0.50**	*1.00*	1.00	0.50
NRG2	9542	65	131	**0.50**	*1.00*	1.00	0.50
ITGA8	8516	66	331	**0.50**	*1.00*	1.00	0.50
MAPK10	5602	67	785	**0.50**	*1.00*	1.00	0.50
GJA1	2697	68	224	*1.00*	**0.50**	1.00	0.50
NCAM1	4684	69	1082	**0.50**	*1.00*	1.00	0.50
SERPINE1	5054	70	593	*1.00*	**0.50**	1.00	0.50

Table S1: Candidate genes associated with autism ($\mathcal{D}$) and cell motility ($\mathcal{P}$), ordered by their combined score $g(v)$. Genes in the top 70 ranking that are unlabeled in either in $\mathcal{D}$ and $\mathcal{P}$ are shown. Italic font indicates that the gene is a positive; bold font indicates that the gene is unlabeled.
